# Chinese Adolescents’ Reading Motivation Profiles and Their Relations to Reading Amount

**DOI:** 10.3389/fpsyg.2022.875486

**Published:** 2022-06-02

**Authors:** Xiaocheng Wang, Yuanying Jin, Lina Jia

**Affiliations:** ^1^Department of Education, School of Humanities, Jiangnan University, Wuxi, China; ^2^Department of Education, School of Humanities, Sejong University, Seoul, South Korea

**Keywords:** profiles, reading motivation, reading amount, Chinese adolescents, latent profile analysis

## Abstract

This study used a person-centered approach to identify reading motivation profiles of 514 Chinese adolescents in seventh- to ninth-grade, based on dimensions of intrinsic reading motivation (curiosity and involvement) and extrinsic reading motivation (grades and competition). Furthermore, the effects of each profile on outcome variables (reading amount for enjoyment and for school) were investigated. Latent profile analyses revealed three reading motivation profiles: *high quantity* (high on all four dimensions), *high intrinsic* (high on curiosity and involvement, low on grades, and competition), and *moderate quantity* (moderate on all four dimensions). The high-intrinsic and high-quantity profiles proved to be equally successful in terms of amount of reading for enjoyment and for school, and both significantly exceeded the moderate-quantity profile. The current findings emphasize the importance of intrinsic reading motivation and the importance of quality of motivation, compared with its quantity.

## Introduction

Reading is considered a fundamental skill that heavily influences future academic achievement and participation in social life. Therefore, fostering both the skill and the will to read, such as reading motivation, has become a central goal in reading instruction (Watkins and Coffey, 2004). The field of motivation research has the potential to extend our understanding of reading development and achievement (Conradi et al., 2014). Previous research on reading motivation has mostly taken a variable-centered approach, examining whole-sample averages and their contributions to outcome variables, such as reading behavior and competence (*cf.* Schiefele et al., 2012). Although variable-centered studies can provide information on the effects of single variables, they largely ignore the fact that variables are organized within individuals (Schiefele and Löweke, 2017) and an outcome such as reading achievement might be the result of a combination of several motives (Pintrich, 2003). The variable-centered approach is limited when it comes to examining complex interactions among variables, particularly when attempting to draw reasonable conclusions about individuals or groups of individuals (Bergman and Magnusson, 1997). Thus, researchers have called for a change to move away from the variable-centered approach and study motivation from a person-centered perspective (e.g., Corpus and Wormington, 2014; Schiefele and Löweke, 2017). In contrast to variable-centered analyses, the person-centered approach examines relationships among variables at the individual level, and then groups individuals who show similar patterns of relationships into a profile (Bergman, 2001). The person-centered perspective can help understand how variations in construct presentation across individuals are associated with outcomes in practical and useful ways (Quirk et al., 2020). In the present study, we used a person-centered approach, i.e., latent profile analysis (LPA; Collins and Lanza, 2010) to identify profiles of Chinese adolescents based on their patterns of reading motivation. We also examined whether significant differences existed among profiles with regard to reading amount. The use of a person-centered perspective in this study will help in complementing the few variable-centered investigations that have been conducted with Chinese students (e.g., Lau, 2004, 2009; Law, 2009; Wang et al., 2020; Wang and Jin, 2021), thereby offering literacy educators and researchers valuable insights into understanding distinctive patterns of student reading motivation and tailoring effective instructional practices.

### Conceptualization and Measurement of Reading Motivation

Reading motivation has been defined as an “individual’s personal goals, values, and beliefs with regard to the topics, processes, and outcomes of reading” (Guthrie and Wigfield, 2000, p. 405). Consistent with general motivation theories, such as the self-determination theory (Deci and Ryan, 1985; Ryan and Deci, 2000), reading motivation is categorized into two broad categories: intrinsic and extrinsic reading motivation. *Intrinsic reading motivation* is defined as the willingness to read because reading is perceived as rewarding or satisfying (e.g., a source of enjoyment) whereas *extrinsic reading motivation* refers to reading because of external values and demands as opposed to reading for its own sake (e.g., a desire for good grades). Although a wide variety of reading motivation scales have been proposed (*cf.* Davis et al., 2018), we strongly recommend the Motivation for Reading Questionnaire (MRQ; Wigfield and Guthrie, 1997; Baker and Wigfield, 1999) that was developed based on various motivation theories (e.g., self-determination theory, self-efficacy theory, and expectancy-value theory) and has been incredibly influential over the last 2 decades (Schiefele et al., 2012; Conradi et al., 2014). Currently, it is considered the most well-established, comprehensive instrument available for measuring reading motivation (Lau, 2004; Schiefele and Schaffner, 2016). The MRQ proposes 11 constructs, grouped into three higher-order categories: *competence beliefs*, *intrinsic and extrinsic reading motivation*, and *social motivation*. In their review of studies concerning the multidimensionality of reading motivation, Schiefele et al. (2012) declared that dimensions of intrinsic and extrinsic reading motivation were genuine motivational constructs, while others indicated either antecedents (e.g., self-efficacy and importance) or consequences (e.g., challenge) of reading motivation.

Although there is only partial agreement on the nature and number of dimensions of intrinsic and extrinsic reading motivation (Schiefele and Schaffner, 2016), researchers seem to have reached a consensus that the following should be regarded as key components: *curiosity* (desire to read about topic of personal interest), *involvement* (enjoyment experienced from reading), *grades* (desire to attain good grades or marks in school), *competition* (desire to outperform peers in reading), and *recognition* (pleasure in receiving recognition for reading success; see Cox and Guthrie, 2001; Wang and Guthrie, 2004; Unrau and Schlackman, 2006; Logan et al., 2011; Schiefele et al., 2012, 2016; McGeown et al., 2016; Schiefele and Schaffner, 2016). Based on our prior works (Wang et al., 2020; Wang and Jin, 2021), we used curiosity and involvement as indicators of intrinsic reading motivation, and grades and competition as indicators of extrinsic reading motivation, in the present study. Different from previous studies (e.g., Guthrie et al., 1999; Cox and Guthrie, 2001; Schiefele and Löweke, 2017), we used grades instead of recognition as a key component of extrinsic reading motivation. This was because recognition is perceived very differently from the other two extrinsic motivational constructs (i.e., grades and competition) and might explain some variance of intrinsic reading motivation in the Chinese cultural context (see Wang et al., 2020 and Wang and Jin, 2021, for more details of this issue).

### Reading Motivation and Reading Amount

Research that applied the MRQ to study reading motivation was mostly variable centered, examining how reading motivation related to outcome variables, such as reading behavior and comprehension (see an overview by Schiefele et al., 2012). Among these variables, reading amount plays an important role in students’ reading development, including benefits in terms of world knowledge, reading comprehension, and social engagement (*cf.* Schiefele et al., 2012). Despite various instruments assessing reading amount, previous research has clearly shown that intrinsic reading motivation is more strongly related to the amount of enjoyment reading, i.e., voluntary reading or reading for pleasure, than extrinsic reading motivation (e.g., Baker and Wigfield, 1999; Wang and Guthrie, 2004; Lau, 2009; Becker et al., 2010). Positive associations between reading amount and intrinsic reading motivation were also confirmed when controlling for other relevant predictors, such as prior knowledge, past reading achievement, extrinsic reading motivation, and reading literacy (e.g., Guthrie et al., 1999; Wang and Guthrie, 2004; Lau, 2009; Becker et al., 2010; Stutz et al., 2016; Kavanagh, 2019; Troyer et al., 2019).

However, evidence regarding the effects of extrinsic reading motivation on reading amount has been mixed. While some studies reported partly positive correlations between extrinsic reading motivation and the amount of reading for enjoyment (e.g., Wigfield and Guthrie, 1997; Baker and Wigfield, 1999; Guthrie et al., 1999), others obtained partly nonsignificant or weak negative effects of extrinsic reading motivation (e.g., Lau, 2009; Becker et al., 2010). Moreover, some researchers revealed a diminished positive effect (or even a negative effect) of extrinsic reading motivation on enjoyment reading amount when controlling for predictors, such as intrinsic reading motivation, prior reading achievement, and reading efficacy (e.g., Wang and Guthrie, 2004; Schaffner et al., 2013). It is suggested that extrinsically motivated readers tend to view reading as a school-related activity instead of an intrinsically rewarding leisure-time activity and only read when they had to (e.g., to achieve better in school; Becker et al., 2010; Schaffner et al., 2013). Thus, the amount of leisure time reading of such readers will not be enhanced or may even be reduced.

With respect to the effects of specific indicators of extrinsic reading motivation, some studies found that grades, competition, recognition, and compliance were positively correlated with the amount of reading for enjoyment (e.g., Wigfield and Guthrie, 1997; Baker and Wigfield, 1999). However, Wang and Guthrie (2004) demonstrated such associations only for competitive reading motivation. Although a comparison of the effects of intrinsic and extrinsic reading motivation on school reading and pleasure reading has not been widely reported, Wang and Guthrie (2004) showed that intrinsic incentives are better predictors than extrinsic incentives, not only in terms of the amount of enjoyment reading, but also in terms of the amount of school reading, with the latter exhibiting either low or no relations with extrinsic reading motivation.

### Profiles of Reading Motivation

Given the scarcity of person-centered research on reading motivation, it is important to look closely at studies referring to a general motivation to learn. Previous person-centered studies focusing on general motivation have mostly used cluster analysis to identify naturally occurring combinations of intrinsic and extrinsic motivations. For example, Corpus and Wormington (2014) classified three motivational profiles among elementary school students: *high quantity* (high intrinsic/high extrinsic), *primarily intrinsic* (high intrinsic/low extrinsic), and *primarily extrinsic* (high extrinsic/low intrinsic). In addition to those found by Corpus and Wormington (2014), Hayenga and Corpus (2010) revealed a *low-quantity* profile (low intrinsic/low extrinsic) of middle school students. Their results were largely consistent with Vansteenkiste et al. (2009) and Wormington et al. (2012), who found similar patterns among high school students. Notably, the primarily intrinsic (also named *good quality*; *cf.* Vansteenkiste et al., 2006, 2009) profiles outperformed or performed as well as high-quantity profiles in a variety of learning outcomes, including self-reported school grades (Vansteenkiste et al., 2009; Hayenga and Corpus, 2010; Wormington et al., 2012), standardized test scores (Corpus and Wormington, 2014), strategy use (Vansteenkiste et al., 2009), and extracurricular activity participation (Wormington et al., 2012). These results echo the self-determination theory that the quality of motivation—the ratio of intrinsic to extrinsic motivation—is more important than the overall amount of motivation present (see Deci and Ryan, 1985; Ryan and Deci, 2000, for more details of this issue).

Regarding motivational profiles in the reading domain, Guthrie et al. (2009) utilized a top-down approach to create reading motivation profiles of fifth graders, based on their patterns of intrinsic motivation, avoidance, self-efficacy, and perceived difficulty. Their process yielded four profiles: *avid readers* (high intrinsic/low avoidance), *ambivalent readers* (high intrinsic/high avoidance), *apathetic readers* (low intrinsic/low avoidance), and *averse readers* (low intrinsic/high avoidance). Expectedly, avid readers showed significantly better scores in reading achievement than all other groups. However, the reading motivation profiles identified and used in the analyses were derived theoretically, rather than empirically.

More recent studies have examined empirically derived reading motivation profiles, utilizing cluster analysis (e.g., Rosenzweig and Wigfield, 2017; Wang, 2021) or LPA (e.g., Schiefele and Löweke, 2017; Jang et al., 2020). For example, Rosenzweig and Wigfield (2017) outlined four profiles of middle school students based on their patterns on self-efficacy, perceived difficulty, value, and devalue of reading: *high affirming-low undermining* (high self-efficacy and value/low perceived difficulty and devalue), *low affirming-high undermining* (low self-efficacy and value/high perceived difficulty and devalue), *high efficacy and devalue* (high self-efficacy and devalue/low perceived difficulty and value), and *moderate* (moderate levels of all variables). Expectedly, the high affirming-low undermining group performed the best, whereas the low affirming-high undermining group performed the worst, on a variety of outcomes including information text comprehension, language arts grades, and dedication to reading.

Schiefele and Löweke (2017) were the only researchers that identified reading motivation profiles based on specific dimensions of intrinsic (involvement and curiosity) and extrinsic (recognition and competition) reading motivation. By means of LPA, they identified four profiles of reading motivation across third- and fourth-grade students: *high intrinsic* (high curiosity and involvement/low recognition and competition), *high involvement* (high involvement/low on the remaining dimensions), *high quantity* (high on all dimensions), and *moderate quantity* (low to moderate on all dimensions). Similar to research referring to general motivation to learn, the two intrinsic profiles (high intrinsic and high involvement) outperformed both the moderate-quantity and high-quantity groups pertaining to sentence and passage comprehension. However, with respect to reading amount, the high-quantity profile was as successful as the two intrinsic profiles.

### Reading Motivation and Reading Amount of Chinese Students

The development of intrinsic and extrinsic reading motivation and their relations to outcome variables are likely to be influenced by an individual’s cultural experiences (Wang and Guthrie, 2004). In a traditional Chinese society, academic success is considered as fundamental to achieve a satisfactory life and obtain a respectable social status (Law, 2011). Therefore, Chinese parents are generally concerned with their children’s achievement since obtaining higher marks at school can lead to future success (Ho, 1986). Within this cultural background, Chinese students generally harbor positive attitudes toward learning and achievement motivation (Stevenson and Lee, 1996). For example, Lau (2004) investigated the motivational aspects of self-efficacy, intrinsic, extrinsic, and social motivation, and attributional belief on reading among seventh graders from Hong Kong. Her results showed that students were highly motivated to read for intrinsic interest and had adaptive attributional beliefs, which accord with those targeting Chinese students from Taiwan (e.g., Wang and Guthrie, 2004; Huang, 2013).

Chinese students from Hong Kong or Taiwan live in a different social and political environment compared with mainland Chinese students. Our prior works (Wang et al., 2020; Wang and Jin, 2021) examined mainland Chinese adolescents’ reading motivation in various dimensions using the MRQ. The results showed that mainland Chinese adolescents characterized themselves as motivated readers with respect to most dimensions. Notably, they scored the highest on intrinsic reading motivation and the lowest on extrinsic reading motivation. Dimensions with higher means included involvement, curiosity, and challenge, whereas the relatively low means included work avoidance, grades, and competition. Moreover, intrinsic reading motivation was positively associated with students’ amount of enjoyment reading while extrinsic reading motivation was not a significant predictor, which is much in line with findings of Western studies.

Although reading is an important component of Chinese language class, independent reading has not been emphasized until very recently (Yi et al., 2018). Influenced by the Confucian heritage culture, traditional Chinese reading instruction mainly follows a teacher-centered and didactic approach (Ho, 2001). Chinese language teachers are used to playing an authoritative role in delivering knowledge and explaining the content of prescribed texts to students (Lau, 2017), with the main goal of teaching students to achieve high scores on standardized tests. Consistently, Lin et al. (2021) indicated that due to the heavy academic burden, teacher-centered instruction, and an unsound campus reading cultural system, Chinese students rarely engage in independent reading in class. This makes their recreational reading and academic reading always mixed at the same time and space (usually outside the classroom). Thus, Chinese students’ reading amount is mainly reflected in out-of-school reading, which consists of both academic and recreational reading.

### The Present Study

Most previous person-centered studies highlighted the intrinsic and extrinsic motivation to learn and read, but failed to differentiate among dimensions within these constructs except for the study by Schiefele and Löweke (2017). While this is the most rigorous study of reading motivation profiles to date, the focus on third- and fourth-grade students from Germany limits the generalizability of the study’s findings in terms of understanding motivation profiles and differences among diverse adolescents from other cultures. Furthermore, although there have been studies that have examined motivational profiles of students from various ethnic backgrounds, almost no study to date has identified motivational profiles specific to Chinese students (for an exception, see Wang, 2021). Therefore, the present study aims to categorize Chinese students based on their intrinsic and extrinsic reading motivation by applying LPA. LPA is a person-centered approach that uses indicator variables to calculate class probabilities for each individual and classifies individuals into classes (Muthén, 2001). It identifies the heterogeneity in the population *via* a model-based clustering approach, that is, a specification of a probabilistic model describing the relationship between the latent profiles and the observed indicators. LPA is considered to have several benefits compared with clustering techniques used in previous studies (Stanley et al., 2017). Specifically, the current study addresses the following research questions:

*Research Question 1*: Which underlying reading motivation profiles are identified among Chinese adolescents?

*Research Question 2*: How is the identified profile membership associated with the amount of reading for enjoyment and for school?

Based on empirical evidence on profiles of general motivation to learn (e.g., Vansteenkiste et al., 2009; Hayenga and Corpus, 2010; Wormington et al., 2012; Corpus and Wormington, 2014), we expected to find four subgroups including two patterns categorized by quantity of motivation (i.e., high quantity and low/moderate quantity), and two categorized by quality of motivation (i.e., high intrinsic and high extrinsic). For the variation in the relationships between profile membership and reading amount, we hypothesized that there would be significant differences in terms of reading amount between different profiles, with high-intrinsic and high-quantity groups reading more, whereas high-extrinsic and low/moderate-quantity groups read less.

## Materials and Methods

### Participants

The present study was part of a survey aiming to examine students’ reading motivation and its relation to reading behavior and achievement (see Wang and Jin, 2020, 2021; Wang et al., 2020; Wang, 2021). Participants were recruited from two public schools in a capital city in Eastern China. We first randomly selected two administrative regions in the city and then selected one school in each area. Both schools follow the national curriculum, and hence teach the same literacy curriculum. The final sample consisted of 514 students (*M*_age_ = 13.30 years, *SD* = 0.97 year), with approximately equal numbers of boys (*n* = 252; 49.0%) and girls (*n* = 262; 51.0%), in seventh- (*n* = 182; 35.4%), eighth- (*n* = 170; 33.1%), and ninth- (*n* = 162; 31.5%) grades. We obtained approval from the schools and maintained sufficient communication with principals, teachers, and parents before data collection. In addition, we informed the participants that all their responses would be kept confidential and used for research purpose only.

### Measures

#### Reading Motivation

Reading motivation was assessed using an abbreviated Chinese version of the MRQ (see Table 1; Wang and Jin, 2021). The MRQ contains four subscales: curiosity (five items), involvement (five items), grades (four items), and competition (four items). The first two subscales referred to intrinsic reading motivation and the other two referred to extrinsic reading motivation. As mentioned earlier, these dimensions were specifically selected because they are considered key components of reading motivation. Students had to rate each item on a Likert scale ranging from 1 (*very different from me*) to 4 (*a lot like me*), with higher scores indicating higher levels of motivation. To examine the construct validity, we tested the fit of a four-factor model (curiosity, involvement, grades, and competition), two-factor model (intrinsic and extrinsic), and second-order four-factor model (nested within intrinsic and extrinsic motivation) using confirmatory factor analysis (CFA). The CFA results indicated that the four-factor model and second-order four-factor model showed a similar level of fit, and both exceeded the two-factor model. Considering the principle of parsimony, the four-factor model was selected as the optimal model. After correlating residuals of items 6 and 7, 13 and 15, and 11 and 13, the four-factor model represented a good fit to the data: χ^2^/*df* = 3.09, comparative fit index = 0.91, Tucker-Lewis index = 0.90, and root mean square error of approximation = 0.06, with each item loading onto the hypothesized motivational scale (see Table 1). The Cronbach’s alpha for the total scale was 0.86, and were 0.78 (curiosity), 0.77 (involvement), 0.73 (grades), and 0.76 (competition) for the four subscales, all falling in the acceptable range.

**Table 1:** The adapted version of Motivation for Reading Questionnaire.

Subscales		Items	Factor loadings
Curiosity (five items, *α* = 0.78)	1.	If the teacher discusses something interesting I might read more about it.	0.59^***^
	2.	I read about my hobbies to learn more about them.	0.69^***^
	3.	I read to learn new information about topics that interest me.	0.72^***^
	4.	I like to read about new things.	0.68^***^
	5.	If I am reading about an interesting topic I sometimes lose track of time.	0.58^***^
Involvement (five items, *α* = 0.77)	6.	I make pictures in my mind when I read.	0.51^***^
	7.	I feel like I make friends with people in good books.	0.59^***^
	8.	I like mysteries.	0.65^***^
	9.	I enjoy a long, involved story or fiction book.	0.75^***^
	10.	I read a lot of adventure stories.	0.62^***^
Grades (four items, *α* = 0.73)	11.	I look forward to finding out my reading grade.	0.66^***^
	12.	Grades are a good way to see how well you are doing in reading.	0.67^***^
	13.	I read to improve my grades.	0.69^***^
	14.	My parents ask me about my reading grade.	0.59^***^
Competition (four items, *α* = 0.76)	15.	I like being the only one who knows an answer in something we read.	0.50^***^
	16.	I try to get more answers right than my friends.	0.75^***^
	17.	I like to finish my reading before other students.	0.77^***^
	18.	I am willing to work hard to read better than my friends.	0.69^***^

*α* = Cronbach’s alpha.

*** *p* < 0.001.

#### Reading Amount

The scale to assess reading amount was adapted from previous instruments (Guthrie et al., 1994; Schaffner et al., 2013). We measured amount of reading in two contexts: reading for personal enjoyment (three items) and reading for school (three items), which were considered two important types of reading for adolescents. For each context, the first two items referred to reading frequency (e.g., “*How many books have you read for interest during the previous month?*”) and the remaining one item captured the length of reading (e.g., “*How long do you usually spend reading a book without taking a break when reading for interest?*”). We exclusively focused on book reading because this aspect of reading amount is clearly more important for the development of reading comprehension than other aspects (e.g., reading on the Internet; *cf.* Pfost et al., 2013). All items were rated on a four-point Likert scale, with higher scores indicating a higher reading amount. The Cronbach’s alpha for the whole scale and for the subscales of enjoyment and school reading amount were 0.66, 0.56, and 0.53, respectively, which is similar to previous research (e.g., Wang and Guthrie, 2004; Stutz et al., 2016). Although the reliabilities were relatively low, the measure was retained considering that it is widely adopted as a measure of reading amount (see also Guthrie et al., 1999; Cox and Guthrie, 2001).

### Analysis

First, data were screened in SPSS 20.0 to check assumptions of normality and to examine descriptive statistics across all variables. Next, we conducted LPAs by using the statistic software Mplus 7.4 (Muthén and Muthén, 1998–2015). We began with a one-profile model, with an increasing number of latent profiles and comparing *k*-profile models with (*k-1*)-profile models iteratively. The best fitting solutions (i.e., number of latent profiles) should involve models coherent with theoretical assumptions, previous findings, and the model fit indices (Marsh et al., 2009). The following indices were used to determine the goodness of fit of the model: log likelihood (LLH), the Akaike information criterion (AIC), the Bayesian information criterion (BIC), and the sample-size-adjusted BIC (ABIC). The optimal profile solution should have the highest LLH value and the lowest AIC, BIC, and ABIC values (Nylund et al., 2007; Nylund-Gibson and Choi, 2018).

To test whether one particular model fits the data significantly better than another one, the Lo–Mendell–Rubin likelihood ratio test (LMRT; Lo et al., 2001) and the bootstrap likelihood ratio test (BLRT; Nylund et al., 2007) were applied. Significant *p* values (<0.05) indicate that the model with *k* profiles is better than a model with *k-*1 profiles, while non-significant *p* values (>0.05) indicate that both models are equally well fitted (Nylund et al., 2007; Nylund-Gibson and Choi, 2018). Additionally, the entropy indicates the precision with which the cases are classified into the profiles (ranging from 0 to 1, with higher values indicating better classification), but should not be used to determine the optimal number of profiles (Asparouhov and Muthén, 2014a).

In addition to model fit indices, we also considered the principle of parsimony in the number of profiles, taking theoretical considerations into account. Furthermore, we identified the percentage of the sample in each profile of a given model. Each profile should include at least 5% of the total sample to provide substantive evidence of each profile (Schiefele and Löweke, 2017; Quirk et al., 2020). However, if a profile contains fewer than 5% of the total sample and represents a substantively different group of participants, it is permissible to use that model based on theoretical considerations (Collins and Lanza, 2010; Schiefele and Löweke, 2017).

Once the optimal number of latent profiles was identified, outcome variables (i.e., enjoyment and school reading amount) were assessed in relation to the profiles by utilizing the DU3STEP procedure in Mplus. The DU3STEP procedure is a three-step approach that determines whether there are statistically significant differences between the profiles on an outcome variable (Asparouhov and Muthén, 2014b,c).

## Results

### Descriptive Statistics and Correlations

Table 2 presents the descriptive statistics of all variables and their intercorrelations. No issues with univariate normality or skewness/kurtosis were identified, and significantly positive correlations between all motivational variables were found. Consistent with expectations, the most positive correlations were found between curiosity and involvement (*r* = 0.53, *p* < 0.01) and between grades and competition (*r* = 0.51, *p* < 0.01). The correlations between motivation variables and reading amount were mostly consistent with previous findings (*cf.* Schiefele et al., 2012). Specifically, intrinsic reading motivation (i.e., curiosity and involvement) was positively associated with amount of reading for enjoyment, whereas extrinsic reading motivation exerted either low (competition) or no (grades) effects. With respect to amount of reading for school, intrinsic reading motivation also showed stronger associations than extrinsic reading motivation. These results also confirmed previous findings showing that intrinsic reading motivation is a stronger predictor of reading amount than extrinsic reading motivation (e.g., Baker and Wigfield, 1999; Wang and Guthrie, 2004; Lau, 2009; Becker et al., 2010) and revealing a positive correlation between amount of enjoyment reading and competition, but not grades (e.g., Wang and Guthrie, 2004; Schiefele and Schaffner, 2016).

**Table 2:** Descriptive statistics and bivariate correlations.

Variable	Mean	*SD*	Skewness	Kurtosis	1	2	3	4	5
1. Curiosity	3.31	0.53	−0.51	0.37	—				
2. Involvement	3.27	0.54	−0.40	−0.25	0.53^**^	—			
3. Grades	2.80	0.67	−0.19	−0.06	0.27^**^	0.31^**^	—		
4. Competition	2.89	0.66	−0.13	−0.17	0.37^**^	0.36^**^	0.51^**^	—	
5. Amount of enjoyment reading	3.00	0.71	−0.27	−0.17	0.32^**^	0.30^**^	0.09	0.11^*^	—
6. Amount of school reading	2.75	0.64	−0.20	0.30	0.29^**^	0.28^**^	0.15^**^	0.14^**^	0.44^**^

* *p* < 0.05;

** *p* < 0.01.

### Identification and Description of the Optimal Profile Solution

Model fit indices of profile solution are presented in Table 3. Specifically, AIC, BIC, and ABIC decreased with each additional profile, indicating a better fit for more complex solutions. Notably, statistical model comparisons using the BLRT were not helpful in this study since all analyses gave values of *p* of <0.001. Hence, the BLRT could not be used for model comparisons (see also Quirk et al., 2020). The value of *p* of the LMRT for the four-profile model was not significant, suggesting that addition of an extra profile to the three-profile model did not provide statistically significant improvements. In addition, the four-profile model contained a profile with a prevalence below 5% (*n* = 12; 2.3%) and did not provide substantive information about profiles of reading motivation in comparison with the three-profile model. In this case, the three-profile model was selected as the most appropriate, which was also more parsimonious. We replicated the three-profile model three times using different starting values and all of them showed the same model fit indices.

**Table 3:** Model fit indices.

Numbers of profiles	Model fit indices							
	LLH	FP	AIC	BIC	ABIC	LMRT *p*-value	BLRT *p*-value	Entropy
1	−1852.747	8	3721.494	3755.431	3730.038	—	—	—
2	−1701.382	13	3428.763	3483.912	3442.648	0.0003	0.0000	0.678
**3**	**−1659.424**	**18**	**3354.848**	**3431.208**	**3374.073**	**0.0452**	**0.0000**	**0.655**
4	−1619.791	23	3285.582	3383.153	3310.147	0.2942	0.0000	0.772

LLH, log likelihood; FP, free parameters; AIC, Akaike information criterion; BIC, Bayesian information criterion; ABIC, sample-size adjusted BIC; LMRT, Lo–Mendell–Rubin likelihood ratio test; and BLRT, bootstrap likelihood ratio test.

The bold-faced values were model fit indices of the final selected model.

LMRT *p*-value, BLRT *p*-value, and entropy are not available for the one-profile model.

**Figure 1:**
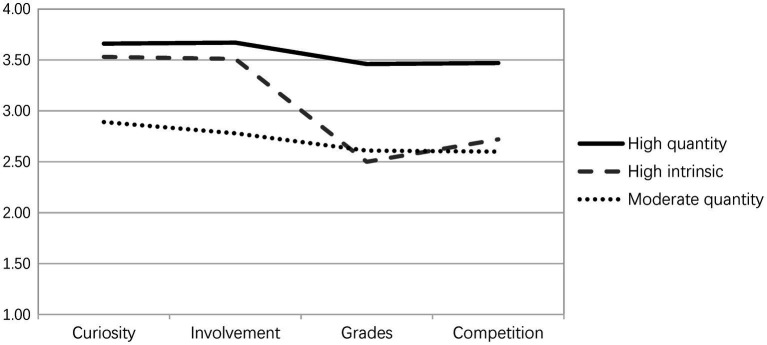
Reading motivation profiles of Chinese adolescents.

Figure 1 presents the identified profiles in the three-profile model (see also Table 4). The *moderate-quantity* profile (*n* = 204; 39.7%) was the largest group identified and was characterized by moderate scores on all four variables (range from 2.60 to 2.89). Students in this group were not highly motivated to read by either intrinsic or extrinsic incentives. The *high-intrinsic* profile (*n* = 161; 31.3%), also called *good-quality* profile (see Vansteenkiste et al., 2009; Hayenga and Corpus, 2010; Wormington et al., 2012), represents students with high levels of curiosity (*M* = 3.53) and involvement (*M* = 3.51) coupled with moderate levels of grades (*M* = 2.50) and competition (*M* = 2.72). Notably, it is characterized by a high ratio of intrinsic to extrinsic reading motivation. The *high-quantity* profile (*n* = 149; 30.0%) was characterized by high scores on all four motivation dimensions (range from 3.46 to 3.66), suggesting that students in this group were both highly intrinsically and extrinsically motivated readers. Compared to the high-intrinsic profile, the high- and moderate-quantity profiles showed balanced ratios of intrinsic to extrinsic reading motivation.

**Table 4:** Descriptive statistics per latent profile.

	Moderate quantity (*n* = 204; 39.7%) *M* (*SD*)	High intrinsic (*n* = 161; 31.3%) *M* (*SD*)	High quantity (*n* = 149; 30.0%) *M* (*SD*)
Curiosity	2.89 (0.05)	3.53 (0.06)	3.66 (0.05)
Involvement	2.78 (0.04)	3.51 (0.07)	3.67 (0.05)
Grades	2.61 (0.06)	2.50 (0.18)	3.46 (0.14)
Competition	2.60 (0.06)	2.72 (0.16)	3.47 (0.11)

*M*, mean; *SD*, standard deviation.

### Relations Between Profile Membership and Reading Amount

Based on previous variable-centered research and person-centered studies concerning general motivation to learn (e.g., Hayenga and Corpus, 2010; Corpus and Wormington, 2014) and read (e.g., Schiefele and Löweke, 2017), we expected the high-intrinsic profile to outperform or at least be equally as successful as the high-quantity profile on reading amount, and both to exceed the moderate-quantity profile. As revealed by Table 5, this assumption was confirmed. The high-intrinsic and high-quantity profiles performed almost equally well, and both significantly exceeded the moderate-quantity profile with respect to amount of reading for enjoyment and for school.

**Table 5:** Profile differences on amount of reading for enjoyment and for school.

Outcome variables	High quantity *M* (*SD*)	High intrinsic *M* (*SD*)	Moderate quantity *M* (*SD*)	*df*	*χ* ^2^
Amount of enjoyment reading	3.25 (0.06)	3.12 (0.08)	2.70 (0.06)	18	46.07^***^
Amount of school reading	2.96 (0.06)	2.85 (0.07)	2.52 (0.05)	18	34.16^***^
*Significant group differences*					
Amount of enjoyment reading	High quantity, high intrinsic > moderate quantity				
Amount of school reading	High quantity, high intrinsic > moderate quantity				

Analyses were run utilizing the DU3STEP procedure in Mplus.

*M*, mean; *SD*, standard deviation.

*** *p* < 0.001.

## Discussion

In this study, we used LPA to identify reading motivation profiles among Chinese adolescents and examined each profile’s level of reading amount for enjoyment and for school. The findings of the present study extend existing variable-centered studies on reading motivation in important ways by employing a person-centered perspective.

### Profiles of Reading Motivation

A three-profile solution was selected as best fitting the data, with groups characterized by high quantity, high intrinsic, and moderate quantity. The profiles mostly replicated the results reported by Schiefele and Löweke (2017), though we could not identify a high-involvement profile as found by Schiefele and Löweke. It seems that Chinese adolescents do not perceive curiosity and involvement differently from each other. This view was also supported by students’ mean levels on curiosity (3.30) and involvement (3.27) and the fact that the highest correlations were found between these two variables (*r* = 0.53, *p* < 0.01; *cf.* Table 2). This result was expected due to the highly impersonal, evaluative, and competitive learning environment in Chinese schools (Lau, 2009), wherein personal interest is generally not considered. Another notable departure was the highest membership in the moderate-quantity profile (39.7%). This was the most populated profile in the current study but was the least populated in the study by Schiefele and Löweke (2017). In their study, most students belonged to the high-intrinsic profile, whereas considerably lower percentages were found in the moderate-quantity profile. This is most likely because they focused explicitly on recreational reading, which is largely a leisure-time activity, and thus tends to depend on intrinsic incentives (Schaffner et al., 2013).

The three-profile solution obtained in this study also somewhat differs from the four-profile solution obtained by studies regarding general motivation to learn (e.g., Vansteenkiste et al., 2009; Hayenga and Corpus, 2010; Wormington et al., 2012; Corpus and Wormington, 2014). Although there was some congruence pertaining to the existence of primarily intrinsic and high-quantity profiles, the low-quantity profile (Vansteenkiste et al., 2009; Hayenga and Corpus, 2010; Wormington et al., 2012) and primarily extrinsic profile (Corpus and Wormington, 2014), also called *poor-quality* profile (*cf.* Vansteenkiste et al., 2009; Hayenga and Corpus, 2010; Wormington et al., 2012), could not be identified in this study. This might be explained by the fact that we used different motivation scales, which may have tapped different types of motivation. The absence of low-quantity and primarily extrinsic groups in our sample is also in accordance with variable-centered research revealing that Chinese students characterized themselves as motivated readers on most dimensions of the MRQ and the lowest score was found for extrinsic reading motivation (see Wang and Jin, 2021).

In the present study, not only was the primarily extrinsic profile absent, but also all identified profiles, including the moderate-quantity profile, were characterized by a favorable ratio of intrinsic to extrinsic reading motivation (see Figure 1). This means that all three profiles have higher levels of intrinsic reading motivation, in different degrees, compared with extrinsic reading motivation. Thus, it can be concluded that the Chinese student sample in the present study was primarily motivated to read by intrinsic incentives, which coincided with students’ mean levels on different motivational dimensions (*cf.* Table 2). Notably, extrinsic reading motivation was only present when there was also a high level of intrinsic reading motivation (e.g., high-quantity; see also Schiefele and Löweke, 2017). Thus, it seems unlikely for Chinese adolescents to read solely for extrinsic reasons. This finding corresponds with claims that Chinese students generally harbor positive attitudes and achievement motivation in learning (e.g., Stevenson and Lee, 1996; Lau, 2004).

### Relations Between Profile Membership and Reading Amount

The observed differences among the profiles in reading amount supported the well-established variable-centered research (e.g., Wigfield and Guthrie, 1997; Wang and Guthrie, 2004; Lau, 2009; Schaffner et al., 2013). Specifically, students with high levels of reading motivation (i.e., high quantity) or higher levels of intrinsic reading motivation (i.e., high intrinsic) outperformed those with lower motivation levels (i.e., moderate quantity) in terms of both enjoyment and school reading amount. The high-intrinsic group performed as well as the high-quantity group despite lacking extrinsic reading motivation. Thus, these results provided good support for the perspective of self-determination theory: the quality of motivation—the ratio of intrinsic to extrinsic motivation—is more important compared with its quantity (see also Vansteenkiste et al., 2009; Hayenga and Corpus, 2010; Wormington et al., 2012; Corpus and Wormington, 2014; Schiefele and Löweke, 2017). Extrinsic motivation did not play a critical role for educational outcomes such as reading amount. The minor role of extrinsic reading motivation also coincided with their low and non-significant correlations with reading amount (*cf.* Table 2). This finding also accords with that of Schiefele and Löweke (2017), who found that the two intrinsic profiles (i.e., high intrinsic and high involvement) as well as the high-quantity profile all exceeded the moderate-quantity profile with respect to reading amount.

However, our results challenge previous findings showing that the high-intrinsic profile displayed the most optimal pattern of educational outcomes relative to all other groups, including the high-quantity profile (e.g., Vansteenkiste et al., 2009; Hayenga and Corpus, 2010). It should be noted that these studies referred to general motivation to learn and did not include reading amount as an outcome variable. Another explanation is that both the high-intrinsic and high-quantity profiles in our study reported high, though slightly different, levels of intrinsic reading motivation compared with levels of extrinsic reading motivation. This means that the high-quantity profile also possessed a clearly favorable ratio of intrinsic to extrinsic reading motivation indicative of “good quality” motivation; these two groups mostly differed in terms of their quantity, but not quality of motivation (Vansteenkiste et al., 2009). However, quantity of motivation does matter when two groups show similar ratios of intrinsic to extrinsic reading motivation, but considerably different amount of motivation (e.g., high-quantity vs. moderate-quantity). Taken together, our results provide further support for previous variable-centered findings showing that intrinsic motivation is the primary correlate of reading amount (e.g., Wang and Guthrie, 2004; Becker et al., 2010; Schaffner et al., 2013). As explained by Schaffner et al. (2013), reading is largely a leisure-time activity, and as such, is strongly controlled by intrinsic incentives.

Simply noting the critical role of intrinsic reading motivation, however, fails to capture nuances of the present study’s findings. If reading amount was driven solely by intrinsic reading motivation, students with a high-quantity profile would have reported the highest reading amount. Instead, the data suggest a compensatory relationship between the ratio of intrinsic to extrinsic reading motivation and the total amount of motivation present. As evidence, the high-quantity profile did not perform better in terms of reading amount than the high-intrinsic profile; although it possessed higher level of intrinsic reading motivation (see Table 4 and Figure 1). Conversely, the high-intrinsic profile was just as adaptive as the high-quantity profile despite possessing less intrinsic reading motivation. This suggests that a high ratio of intrinsic to extrinsic reading motivation probably compensates for a relatively low total amount of motivation (e.g., high-intrinsic), which aligns with claims about the benefits of intrinsic motivation compared with extrinsic motivation (Deci and Ryan, 1985). The presence of extrinsic reading motivation seems to yield no benefit at all and may even undermine the positive effects of intrinsic reading motivation. It thus might be concluded that perhaps the absence of extrinsic reading motivation is more critical than the presence of intrinsic reading motivation in directing the relation between motivation and outcome variables such as reading amount. This pattern of results echoes the findings of person-centered studies concerning general motivation to learn (e.g., Hayenga and Corpus, 2010; Wormington et al., 2012; Corpus and Wormington, 2014).

Given the academic nature of school reading, one might imagine that the high-quantity profile, with high levels of both intrinsic and extrinsic reading motivation, would perform better than the high-intrinsic profile regarding amount of school reading. However, there were no differences between these two profiles in predicting students’ amount of reading for school. This suggests that an addition of extrinsic reading motivation did not exert any positive effect on amount of school reading. This view was supported by correlations between school reading amount and intrinsic and extrinsic reading motivation (*cf.* Table 2; see also Wang and Guthrie, 2004). As mentioned earlier, due to heavy learning pressure, the teacher-centered instruction, and an unsound campus reading cultural system, Chinese students rarely read independently in class. Instead, they usually carry out academic reading activities outside of the classroom (Lin et al., 2021). In this case, their recreational reading and academic reading may always be mixed and happen at the same time and in the same space (usually out of school). Thus, these two types of reading may not be easily differentiated.

### Limitations and Future Research

While the present study provided evidence for meaningful motivational profiles and their relevance for reading amount and underscored the importance of studying reading motivation using a person-centered approach, several limitations must be acknowledged. First, this study was conducted with samples from two schools in eastern China, where the school system, educational culture and conditions might be different from those of other regions. The relatively small sample leads us to view the present results as preliminary findings that should be investigated with broader and more diverse samples. Moreover, focusing on Chinese sample results in limited generalizability of the present findings to other cultures.

Second, the use of LPA includes somewhat subjective decisions (Collins and Lanza, 2010). For example, identifying the optimal number of profiles was based on comparative and not absolute fit indices. Furthermore, as one of the important indices, the entropy did not exceed 0.70 (*cf.* Meeus et al., 2011). Thus, additional criteria, such as interpretability and practicality (Logan and Pentimonti, 2016) and parsimony of profile models, must be considered. Moreover, replication studies are highly desirable.

Third, we used only four dimensions of the MRQ which were considered key components of intrinsic and extrinsic reading motivation. It is possible that more profiles would emerge if more motivational variables were used. To obtain a more complete understanding of profiles of Chinese students’ reading motivation, the 11 dimensions under three categories of *efficacy beliefs*, *intrinsic and extrinsic reading motivation*, and *social motivation*, should all be included and tested in future research. For example, since social motivation seems to be particularly important to Chinese students who are socialized under a collectivistic culture (Yang, 1997), and adolescents are concerned with peer social perception (Juvonen, 1996), it would be interesting to re-examine the motivational profiles as well as their academic correlates after including variables of social motivation. If more subtle differences in motivational profiles emerged, it would provide additional empirical evidence for understanding Chinese adolescents’ reading motivation and tailoring more effective instructional practices. In addition, the identified profiles were evaluated in association with outcome variables, i.e., reading amount for enjoyment and for school, which showed relatively low reliabilities and thus needed to be re-examined in future research. Moreover, other relevant outcome variables, such as reading comprehension and strategy use, also need to be considered.

Finally, longitudinal assessments of reading motivations are absent from this study. Future research should examine the temporal stability of profiles within a single sample and investigate whether some students might change to another profile because of being exposed to a particular intervention or teaching environment. Such research might be an important step toward designing more effective interventions that could better engage students and facilitate their literacy learning.

Despite the limitations discussed above, the findings of this study represent a significant contribution to the current reading motivation research literature, as it was the first study of its kind to examine the reading motivation of Chinese adolescents from a person-centered perspective. This study sheds light on previous findings from variable-centered studies of reading motivation and provides a richer understanding of how adolescents with different motivational profiles perform in terms of reading amount. Specifically, our results suggest that the high-intrinsic profile, which is mostly motivated by curiosity and involvement while less motivated by grades and competition, is as successful as the high-quantity profile which is motivated by both intrinsic and extrinsic incentives. Intrinsic reading motivation seems to show more positive effects if extrinsic reading motivation is low. Therefore, the findings support earlier guidelines for educational practice that focused on fostering interest and enjoyment in reading while avoiding extrinsic incentives (e.g., Guthrie et al., 2004). Such intervention seems to be particularly important especially considering that the largest number of our sample displayed a profile with only moderate levels of reading motivation. Future research should also examine relevant context factors that may impact students’ reading motivation (e.g., Eccles and Wigfield, 2000) and design interventions that alleviate the general decline of intrinsic motivation at the secondary school level.

## Data Availability Statement

The raw data supporting the conclusions of this article will be made available by the authors, without undue reservation, to any qualified researcher.

## Ethics Statement

The studies involving human participants were reviewed and approved by the Ethics Committee of Jiangnan University. Written informed consent to participate in this study was provided by the participants’ legal guardian/next of kin.

## Author Contributions

XW designed the study and was responsible for the statistical analyses and the writing of the manuscript. YJ and LJ provided ideas for data analysis and manuscript writing. All authors contributed to the article and approved the submitted version.

## Funding

This study was supported by the National Social Science Fund of China (CHA150180) and the High-Level Innovation and Entrepreneurship Talents Introduction Program of Jiangsu Province of China.

## Conflict of Interest

The authors declare that the research was conducted in the absence of any commercial or financial relationships that could be construed as a potential conflict of interest.

## Publisher’s Note

All claims expressed in this article are solely those of the authors and do not necessarily represent those of their affiliated organizations, or those of the publisher, the editors and the reviewers. Any product that may be evaluated in this article, or claim that may be made by its manufacturer, is not guaranteed or endorsed by the publisher.
